# (3,5-Di­methyl­adamantan-1-yl)ammonium methane­sulfonate (memanti­nium mesylate): synthesis, structure and solid-state properties

**DOI:** 10.1107/S2056989019009988

**Published:** 2019-07-26

**Authors:** Mihaela Tuksar, Mirta Rubčić, Ernest Meštrović

**Affiliations:** a PLIVA Croatia Ltd., TAPI Research and Development, Prilaz baruna Filipovića 29, HR-10000 Zagreb, Croatia; b Department of Chemistry, Faculty of Science, University of Zagreb, Horvatovac 102a, HR-10000 Zagreb, Croatia

**Keywords:** crystal structure, memantine, X-ray diffraction, differential scanning calorimetry (DSC), thermogravimetric analysis (TGA), IR spectroscopy

## Abstract

The asymmetric unit of the title compound, C_12_H_22_N^+^·CH_3_O_3_S^−^, consists of three (3,5-di­methyl­adamantan-1-yl)ammonium cations, C_12_H_22_N^+^, and three methane­sulfonate anions, CH_3_O_3_S^−^. In the crystal, the cations and anions associate *via* N—H⋯O hydrogen bonds into layers, parallel to the (001) plane, which include large supra­molecular hydrogen-bonded rings.

## Abstract 2

The title salt crystallizes with three independent ionic pairs in the asymmetric unit. In the crystal, (3,5-di­methyl­adamantan-1-yl)ammonium cations and methane­sulfonate anions associate *via* N—H⋯O hydrogen bonds into layers that extend parallel to (001) and comprise large supra­molecular hydrogen-bonded rings.

## Chemical context   

Memantine or 3,5-di­methyl­adamantane-1-yl­amine is an active pharmaceutical ingredient which acts as an uncompetitive NMDA receptor antagonist (Reisberg *et al.*, 2003 ▸; Rammes *et al.*, 2008 ▸; Parsons *et al.*, 2013 ▸). The compound was approved for the treatment of moderate-to-severe Alzheimer’s disease and is currently marketed as the chloride salt. The crystal structure of memanti­nium chloride 0.1-hydrate has previously been described (Lou *et al.*, 2009 ▸). Herein we report the structure of an alternative salt, (3,5-di­methyl­adamantan-1-yl)ammonium methane­sulfonate (I) (memanti­nium mesylate), developed with the aim of producing a material with physico-chemical properties superior to those of memanti­nium chloride.
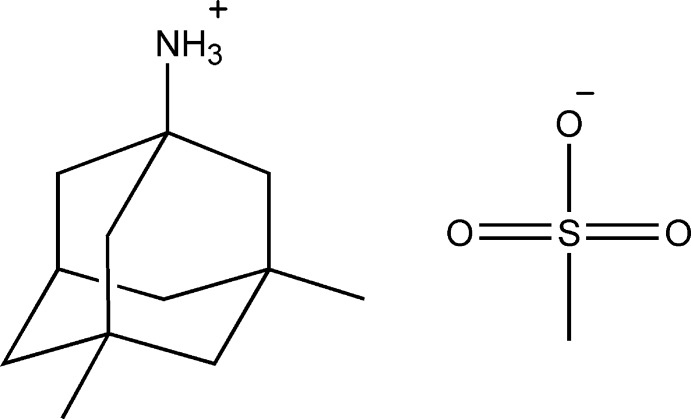


## Structural commentary   

**Figure 1:**
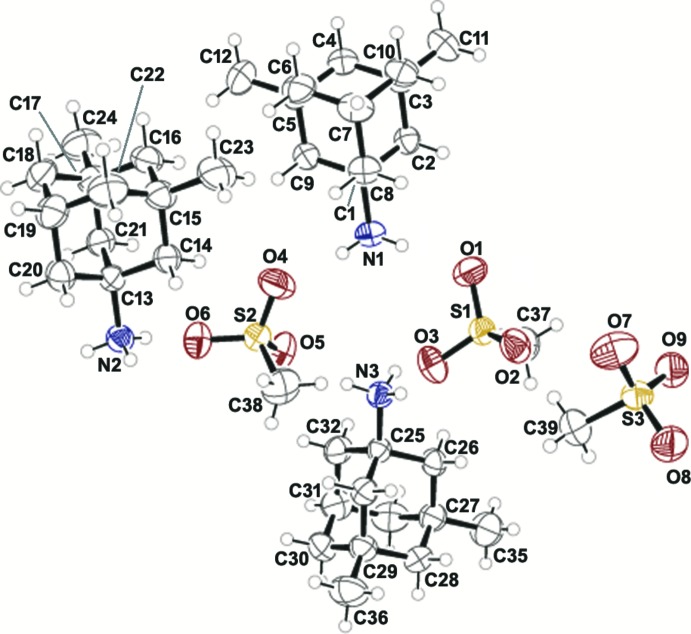
*ORTEP* plot of the title compound. Displacement ellipsoids are drawn at the 50% probability level and H atoms are shown as spheres of arbitrary small radii.

The asymmetric unit of (3,5-di­methyl­adamantan-1-yl)ammonium methane­sulfonate, (I) (Fig. 1 ▸) consists of three crystallographically independent (3,5-di­methyl­adamantan-1-yl)ammonium cations and three methane­sulfonate anions. The structure of the cations is rigid, with all four six-membered rings of the adamantane core of the (3,5-di­methyl­adamantan-1-yl)ammonium cations assuming a typical chair conformation. No significant geometrical differences are observed between the independent cations, or between the methane­sulfonate anions. The (3,5-di­methyl­adamantan-1-yl)ammonium cations are achiral. They possess a plane of symmetry by which two enanti­omorphic halves of the ion, containing chiral centers (C3 and C5, C15 and C17, C27 and C29), are reflections of each other.

## Supra­molecular features   

**Figure 2:**
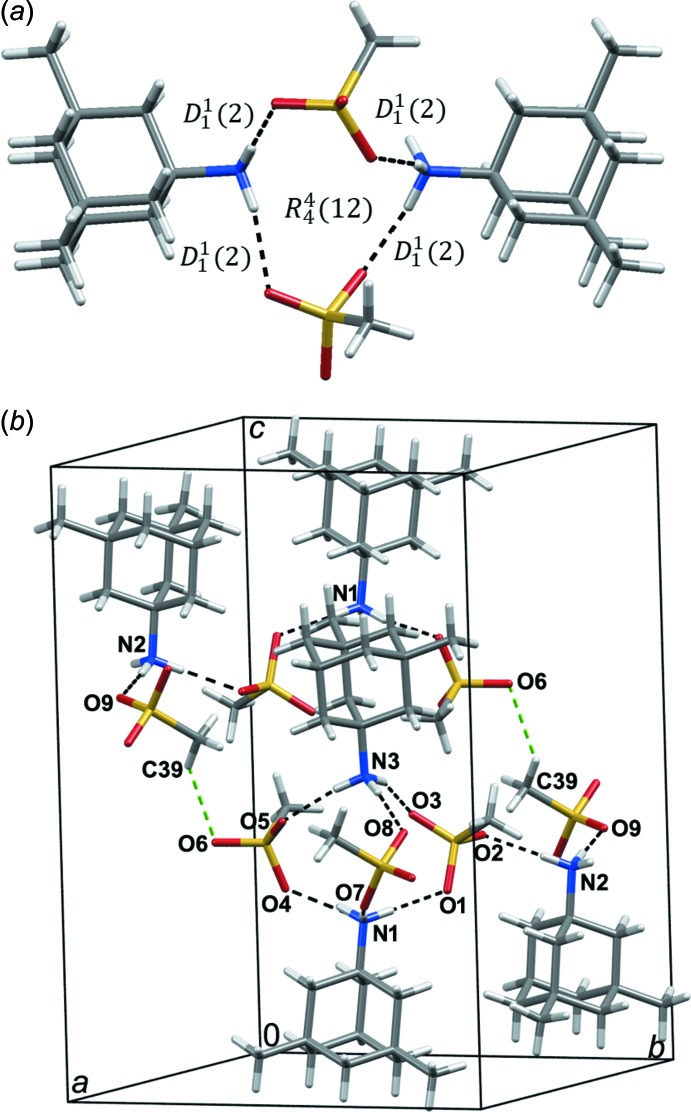
(*a*) A view of the *D*_1_^1^(2) and 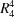
(12) motifs formed *via* N—H⋯O hydrogen bonds. (*b*) Crystal packing of the title compound showing relevant hydrogen bonds and C—H⋯O contacts. Hydrogen bonds are indicated by black dashed lines, while the C—H⋯O contacts are shown as green dashed lines.

**Figure 3:**
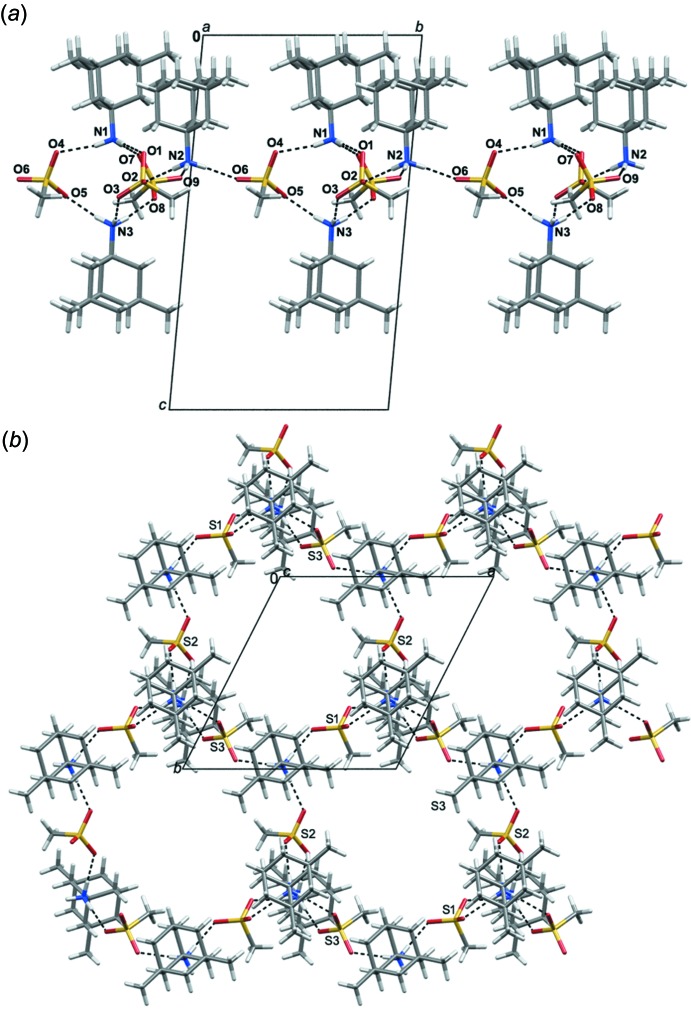
Crystal packing of the title compound showing the layers parallel to (001) based on hydrogen bonded rings. View of the structure: (*a*) along the [100] direction; (*b*) along the [001] direction. Hydrogen bonds are indicated by dashed lines.

The crystal packing of the title compound is characterized by hydrogen-bonding inter­actions between the protonated amino groups of cations and the oxygen atoms of the methane­sulfonate anions (Table 1 ▸, Fig. 2 ▸). Each hydrogen atom of the protonated amino groups of the (3,5-di­methyl­adamantan-1-yl)ammonium cations is engaged in hydrogen bonding with the neighbouring methane­sulfonate anions. While each of the established N—H⋯O hydrogen bonds has a characteristic *D*_1_^1^(2) graph-set motif, they combine into larger 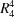
(12) motifs (Fig. 2 ▸). Assemblies formed in such a way are supported by weaker C—H⋯O contacts, as shown in Fig. 2 ▸. Such connectivity leads to the formation of supra­molecular layers parallel to the (001) plane, which involve large hydrogen-bonded rings (Fig. 3 ▸).

## Database survey   

A search of the Cambridge Structural Database (CSD version 5.40, update of November 2018; Groom *et al.*, 2016 ▸) for structures containing the (3,5-di­methyl­adamantan-1-yl)ammonium cation gave four hits: (3,5-dimethyl-1-adamant­yl)ammonium chloride hydrate (DUCYAC; Lou *et al.*, 2009 ▸), 3,5-di­methyl­adamantane-1-ammonium cucurbit[8]uril chloride hexa­cosa­hydrate (GAWLIC, Hostaš *et al.*, 2016 ▸), cucurbit[7]uril memantine clathrate chloride hydrate (SULZIJ, McInnes *et al.*, 2010 ▸) and 3,5-di­methyl­adamantan-1-yl­ammonium 2,4,6-triiso­propyl­benzene­sulfonate (YECDIW, Tkachev *et al.*, 2017 ▸). Among these, the structure of 3,5-di­methyl­adamantan-1-yl­ammonium 2,4,6-triiso­propyl­benzene­sulfonate shows the greatest similarity in its hydrogen-bonding motifs with those observed in the title compound. In the structure of YECDIW, N—H⋯O hydrogen bonds having a *D*_1_^1^(2) graph-set motif dominate the crystal packing. However, in contrast to the hydrogen-bonded layers in the title structure, a complex chain-like hydrogen-bonding network is formed. Such differences can be attributed, at least to some extent, to the distinct steric demands of the anions present in these structures.

## Hirshfeld surface analysis   

**Figure 4:**
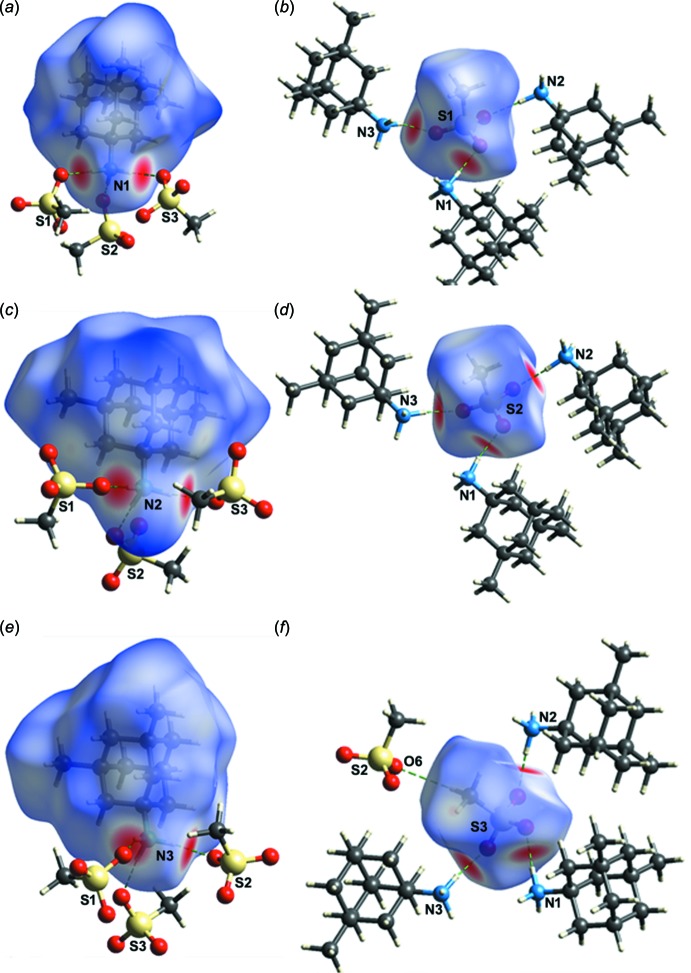
Views of the Hirshfeld surfaces mapped over *d*_norm_ for: (*a*) the N1-containing cation; (*b*) the S1-containing anion, (*c*) the N2-containing cation; (*d*) the S2-containing anion, (*e*) the N3-containing cation and (*f*) the S3-containing anion (range: −0.6178 to 1.7852 a.u.).

**Figure 5:**
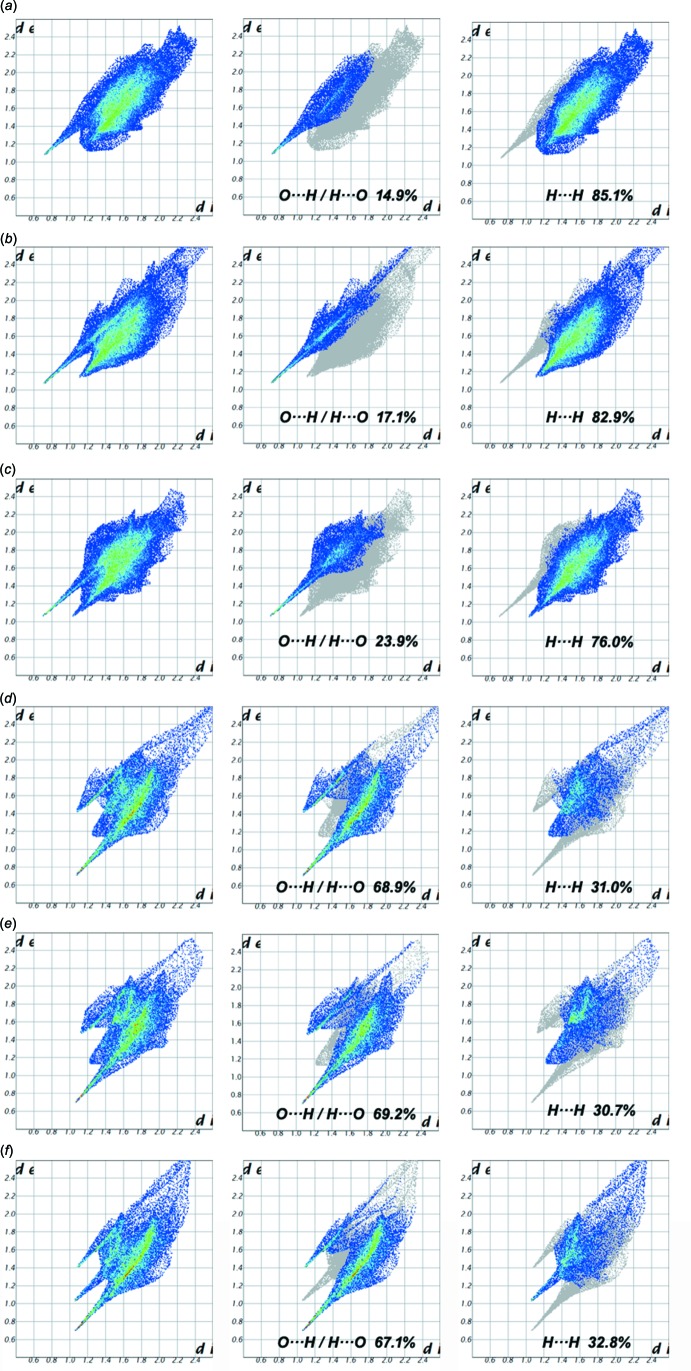
The fingerprint plots for the ions constituting the asymmetric unit of (I): (*a*) the N1-containing cation; (*b*) the N2-containing cation, (*c*) the N3-containing cation; (*d*) the S1-containing anion, (*e*) the S2-containing anion and (*f*) the S3-containing anion. Left side: full fingerprint plot, middle: contribution of the H⋯O/O⋯H contacts, and right side: contribution of the H⋯H contacts to the inter­molecular inter­actions.

The Hirshfeld surfaces for the cations and anions constituting the asymmetric unit of (I) were calculated using *CrystalExplorer17* (Turner *et al.*, 2017 ▸) and are shown in Fig. 4 ▸. Mapping the *d*_norm_ values on the corresponding Hirshfeld surface allows a detailed analysis of hydrogen bonds and short inter­molecular contacts (Spackman & Jayatilaka, 2009 ▸). In this case, red spots indicate N—H⋯O hydrogen bonds, blue regions correspond to positive *d*_norm_ values, and white areas indicate contacts of equal length to the sum of the van der Waals radii, *i.e. d*_norm_ is 0. While the Hirshfeld surfaces for the three cations appear similar to each other, the two-dimensional fingerprint plots reveal distinctive differences between them. The full two-dimensional fingerprint plots along with the decomposed ones, displaying the contributions of the relevant contacts, are shown in Fig. 5 ▸. It can be seen that the N3-containing cation has the largest contribution of H⋯O/O⋯H contacts (23.9%), while for the N1- and N2-containing cations this contribution amounts to 14.9 and 17.1%, respectively. Analysis of the fingerprint plots for the anions reveals that they have fairly similar environments within the crystal and consequently a comparable distribution of the inter­molecular contacts (Fig. 5 ▸).

## Synthesis and crystallization   

To a solution of 10.0 g of (3,5-di­methyl­adamantan-1-yl)ammonium chloride (supplied by PLIVA Croatia Ltd.) in 300 ml of water, 140 ml of toluene was added and the pH adjusted to about 10.7 by using 40% NaOH (aq). The toluene and water layers were separated. To the toluene solution of 3,5-di­methyl­adamantane-1-yl­amine, 3.3 ml of methane­sulfonic acid at 293–298 K was added. The reaction mixture was stirred at 293–298 K for 1 h, cooled to 273–278 K and stirred at that temperature for 1 h. The resulting crystals were filtered off, washed with toluene and dried at 313 K/20 mbar for about 15 h. The obtained solid was slurried in 125 ml of acetone at 293–298 K for about 18 h, filtered off, washed with acetone and dried at 313 K/20 mbar for about 15 h. The product was recrystallized from *i*-propyl acetate, yielding crystals suitable for single-crystal X-ray diffraction, yield 11.7 g (92%).

## Thermal analysis   

The thermal stability of the title compound was investigated in the solid state by thermogravimetric analysis (TGA) and by differential scanning calorimetry (DSC). Thermogravimetric analysis was performed on TA Instruments TGA in closed aluminium pans with one hole on the crucible under a nitro­gen flow (50 mL min^−1^) with a heating rate of 10°C min^−1^ in the temperature range 25–300°C.

**Figure 6:**
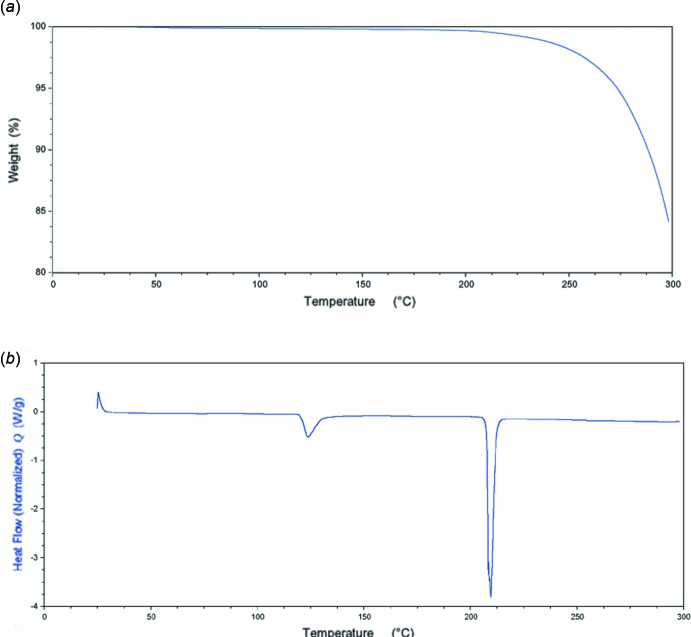
(*a*) TG curve of (I); (*b*) DSC curve of (I).

**Figure 7:**
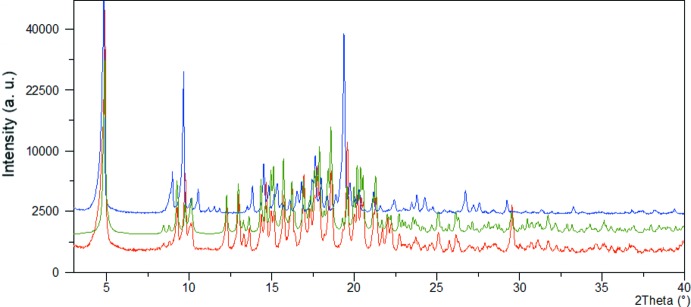
PXRD pattern of the bulk sample of I (red), simulated pattern for (I) (green), and PXRD pattern of the new phase obtained by heating (I) at about 130°C for 17 h (blue).

Thermogravimetric analysis does not reveal any weight loss during heating up to about 200°C, whereupon a change in mass is observed that can be associated with the thermal decomposition of the sample (Fig. 6 ▸ *a*). DSC analysis of (I) reveals two thermal events (Fig. 6 ▸ *b*). The first endotherm at about 125°C suggests that the sample is experiencing a phase transition, as no weight loss can be observed on the corresponding TG curve in this temperature region. The second strong endotherm, observed on the DSC curve at about 210°C, can be ascribed to the melting point of the new phase. Existence of a new, stable phase was confirmed *via* a PXRD experiment, where comparison of the powder patterns of the starting sample (I) and the one obtained by heating (I) at about 130°C for 17 h revealed significant differences (Fig. 7 ▸). Additional confirmation for this conclusion is found in the DSC curve of the material obtained after heating (I), where only one endothermic event can be observed, the one appearing at 210°C and corresponding to its melting point.

## IR spectroscopy   

**Figure 8:**
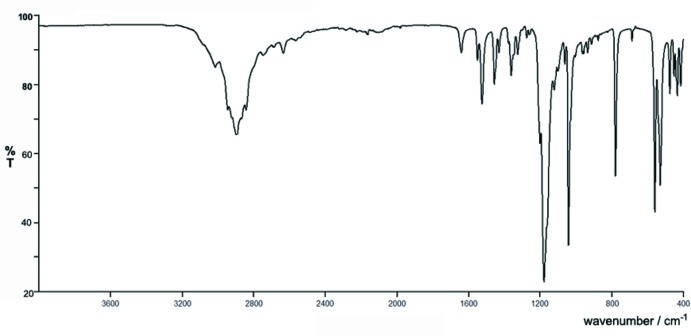
IR spectrum of the title compound.

The infrared (IR) spectrum of title compound was recorded by using the ATR (attenuated total reflectance) technique on a PerkinElmer Spectrum Two instrument. The spectrum of (I) displays a broad band positioned at *ca* 2900 cm^−1^, which corresponds to N—H stretching vibrations of the protonated amino group of the (3,5-di­methyl­adamantan-1-yl)ammonium cations superimposed with the C—H stretching vibrations of the adamantane skeleton and methyl groups of the methane­sulfonate anion (Fig. 8 ▸). The bands corresponding to the S—O asymmetric and symmetric stretching modes appear at 1179 and 1042 cm^−1^, respectively (Başköse *et al.*, 2012 ▸). The band at 780 cm^−1^ is associated with the C—S stretching vibration, whereas the one at 540 cm^−1^ corresponds to the bending mode of the SO_3_ moiety (Başköse *et al.*, 2012 ▸).

## Refinement   

Crystal data, data collection and structure refinement details are summarized in Table 2 ▸. Hydrogen atoms bonded to carbon atoms of the adamantane core were refined as riding with C—H = 0.98 Å for methine C atoms (C7—H7, C19—H19 and C31—H31) and C—H = 0.97 Å for the methyl­ene H atoms, both with *U*_iso_(H) = 1.2*U*_eq_(C). Hydrogen atoms bonded to carbon atoms of the methyl groups of both the memantine cations and the methane­sulfonate anions were refined as rotating rigid groups with C—H = 0.96 Å and *U*_iso_(H) = 1.5*U*_eq_(C). Hydrogen atoms bonded to nitro­gen atoms were found in the difference-Fourier maps at final steps of the refinement and refined with *U*_iso_(H) = 1.2*U*_eq_(N). Their coordinates were refined independently, but N—H distances were restrained to 0.89 (2) Å.

## Supplementary Material

Crystal structure: contains datablock(s) I. DOI: 10.1107/S2056989019009988/fy2137sup1.cif

Structure factors: contains datablock(s) I. DOI: 10.1107/S2056989019009988/fy2137Isup2.hkl

TOC graphic. DOI: 10.1107/S2056989019009988/fy2137sup3.tif

Supporting information file. DOI: 10.1107/S2056989019009988/fy2137Isup4.cml

CCDC reference: 1942388

Additional supporting information:  crystallographic information; 3D view; checkCIF report

## Figures and Tables

**Table 1:** Hydrogen-bond geometry (Å, °)

*D*—H⋯*A*	*D*—H	H⋯*A*	*D*⋯*A*	*D*—H⋯*A*
N1—H1*A*⋯O7^i^	0.90 (2)	1.92 (2)	2.819 (2)	177 (2)
N1—H1*B*⋯O1	0.90 (2)	1.94 (2)	2.833 (2)	179 (2)
N1—H1*C*⋯O4	0.89 (2)	1.96 (2)	2.844 (2)	170 (2)
N2—H2*A*⋯O2^ii^	0.88 (2)	1.92 (2)	2.7991 (18)	179 (2)
N2—H2*B*⋯O9^ii^	0.87 (2)	1.94 (2)	2.8090 (19)	175 (2)
N2—H2*C*⋯O6	0.90 (2)	1.90 (2)	2.7923 (19)	177 (2)
N3—H3*A*⋯O3	0.90 (2)	1.91 (2)	2.7717 (19)	159 (2)
N3—H3*B*⋯O5	0.90 (2)	1.89 (2)	2.7752 (19)	172 (2)
N3—H3*C*⋯O8^i^	0.89 (2)	1.90 (2)	2.785 (2)	172 (2)
C39—H39*B*⋯O6^iii^	0.96	2.59	3.423 (3)	145

Symmetry codes: (i) 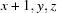; (ii) 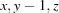; (iii) 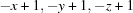.

**Table 2:** Experimental details

Crystal data	
Chemical formula	C_12_H_22_N^+^·CH_3_O_3_S^−^
*M*_r_	275.40
Crystal system, space group	Triclinic, *P* 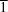
Temperature (K)	295
*a*, *b*, *c* (Å)	11.7761 (2), 11.8731 (2), 18.2788 (3)
α, β, γ (°)	92.501 (2), 94.696 (2), 116.609 (2)
*V* (Å^3^)	2268.09 (8)
*Z*	6
Radiation type	Cu *K*α
μ (mm^−1^)	1.92
Crystal size (mm)	0.32 × 0.21 × 0.11

Data collection	
Diffractometer	Oxford Diffraction Xcalibur Sapphire3
Absorption correction	Multi-scan (*CrysAlis PRO*; Rigaku, 2018 ▸)
*T*_min_, *T*_max_	0.200, 1.000
No. of measured, independent and observed [*I* > 2σ(*I*)] reflections	76762, 8992, 8048
*R*_int_	0.062
(sin θ/λ)_max_ (Å^−1^)	0.620

Refinement	
*R*[*F* ^2^ > 2σ(*F* ^2^)], *wR*(*F* ^2^), *S*	0.047, 0.133, 1.06
No. of reflections	8992
No. of parameters	523
No. of restraints	9
H-atom treatment	H atoms treated by a mixture of independent and constrained refinement
Δρ_max_, Δρ_min_ (e Å^−3^)	0.86, −0.53

Computer programs: *CrysAlis PRO* (Rigaku, 2018 ▸), *SHELXT* (Sheldrick, 2015*a* ▸), *SHELXL2018* (Sheldrick, 2015*b* ▸) and *Mercury* (Macrae *et al.*, 2008 ▸).

## supplementary crystallographic information

### Crystal data

**Table:**

C_12_H_22_N^+^·CH_3_O_3_S^−^	*Z* = 6
*M**_r_* = 275.40	*F*(000) = 900
Triclinic, *P*1	*D*_x_ = 1.210 Mg m^−^^3^
*a* = 11.7761 (2) Å	Cu *K*α radiation, λ = 1.54184 Å
*b* = 11.8731 (2) Å	Cell parameters from 30823 reflections
*c* = 18.2788 (3) Å	θ = 5.1–72.7°
α = 92.501 (2)°	µ = 1.92 mm^−^^1^
β = 94.696 (2)°	*T* = 295 K
γ = 116.609 (2)°	Prism, colorless
*V* = 2268.09 (8) Å^3^	0.32 × 0.21 × 0.11 mm

### Data collection

**Table:**

Oxford Diffraction Xcalibur Sapphire3 diffractometer	8992 independent reflections
Radiation source: fine-focus sealed X-ray tube, Enhance (Cu) X-ray Source	8048 reflections with *I* > 2σ(*I*)
Graphite monochromator	*R*_int_ = 0.062
Detector resolution: 16.1285 pixels mm^-1^	θ_max_ = 72.9°, θ_min_ = 4.2°
ω scans	*h* = −13→14
Absorption correction: multi-scan (CrysAlis PRO; Rigaku, 2018)	*k* = −14→14
*T*_min_ = 0.200, *T*_max_ = 1.000	*l* = −22→22
76762 measured reflections	

### Refinement

**Table:**

Refinement on *F*^2^	Primary atom site location: dual
Least-squares matrix: full	Hydrogen site location: mixed
*R*[*F*^2^ > 2σ(*F*^2^)] = 0.047	H atoms treated by a mixture of independent and constrained refinement
*wR*(*F*^2^) = 0.133	*w* = 1/[σ^2^(*F*_o_^2^) + (0.0822*P*)^2^ + 0.429*P*] where *P* = (*F*_o_^2^ + 2*F*_c_^2^)/3
*S* = 1.06	(Δ/σ)_max_ < 0.001
8992 reflections	Δρ_max_ = 0.86 e Å^−^^3^
523 parameters	Δρ_min_ = −0.53 e Å^−^^3^
9 restraints	

### Special details

**Table:**

Geometry. All esds (except the esd in the dihedral angle between two l.s. planes) are estimated using the full covariance matrix. The cell esds are taken into account individually in the estimation of esds in distances, angles and torsion angles; correlations between esds in cell parameters are only used when they are defined by crystal symmetry. An approximate (isotropic) treatment of cell esds is used for estimating esds involving l.s. planes.

### Fractional atomic coordinates and isotropic or equivalent isotropic displacement parameters (Å^2^)

**Table:**

	*x*	*y*	*z*	*U*_iso_*/*U*_eq_	
S1	0.63651 (4)	0.78463 (4)	0.39086 (2)	0.04527 (12)	
S2	0.67423 (4)	0.34333 (4)	0.38657 (2)	0.04606 (12)	
S3	0.12114 (4)	0.83219 (4)	0.39052 (2)	0.04759 (12)	
O2	0.51832 (13)	0.79458 (13)	0.39206 (8)	0.0608 (3)	
O6	0.66922 (13)	0.21916 (12)	0.38512 (8)	0.0615 (3)	
O3	0.63630 (13)	0.68285 (13)	0.43196 (8)	0.0637 (4)	
O9	0.22683 (12)	0.95327 (12)	0.38131 (8)	0.0597 (3)	
N2	0.46247 (14)	−0.01597 (13)	0.33925 (7)	0.0433 (3)	
H2A	0.4793 (18)	−0.0755 (16)	0.3563 (11)	0.052*	
H2B	0.3914 (16)	−0.0237 (19)	0.3549 (11)	0.052*	
H2C	0.5300 (16)	0.0593 (15)	0.3524 (11)	0.052*	
N3	0.84089 (14)	0.66545 (13)	0.50755 (7)	0.0443 (3)	
H3A	0.7713 (16)	0.6768 (19)	0.4943 (11)	0.053*	
H3B	0.8237 (19)	0.5892 (15)	0.4864 (11)	0.053*	
H3C	0.9054 (17)	0.7263 (17)	0.4886 (11)	0.053*	
O5	0.78864 (13)	0.43837 (13)	0.42985 (9)	0.0681 (4)	
O8	0.03123 (14)	0.84536 (14)	0.43564 (9)	0.0701 (4)	
O1	0.66929 (16)	0.77755 (15)	0.31635 (8)	0.0689 (4)	
N1	0.79465 (15)	0.63192 (15)	0.27752 (8)	0.0492 (3)	
H1A	0.8792 (15)	0.6704 (19)	0.2924 (11)	0.059*	
H1B	0.7552 (19)	0.6784 (19)	0.2896 (11)	0.059*	
H1C	0.7541 (19)	0.5562 (16)	0.2945 (11)	0.059*	
O4	0.65613 (18)	0.37874 (16)	0.31366 (8)	0.0799 (4)	
O7	0.06024 (15)	0.75862 (18)	0.32090 (8)	0.0807 (5)	
C1	0.78006 (15)	0.61136 (14)	0.19499 (8)	0.0403 (3)	
C13	0.44439 (15)	−0.03377 (14)	0.25673 (8)	0.0410 (3)	
C21	0.57125 (16)	−0.00684 (16)	0.22910 (9)	0.0466 (3)	
H21A	0.602030	−0.063954	0.248806	0.056*	
H21B	0.633874	0.079104	0.245897	0.056*	
C2	0.85215 (15)	0.73767 (14)	0.16319 (9)	0.0437 (3)	
H2D	0.941858	0.775209	0.182302	0.052*	
H2E	0.818326	0.795085	0.177964	0.052*	
C25	0.86750 (14)	0.67267 (14)	0.58989 (8)	0.0388 (3)	
C8	0.63814 (16)	0.55422 (17)	0.16706 (9)	0.0495 (4)	
H8A	0.603732	0.611128	0.181834	0.059*	
H8B	0.591700	0.474521	0.187850	0.059*	
C14	0.39642 (17)	0.05787 (16)	0.22769 (9)	0.0465 (4)	
H14A	0.458103	0.144183	0.244420	0.056*	
H14B	0.316197	0.041308	0.246425	0.056*	
C33	0.74579 (15)	0.58585 (15)	0.62134 (9)	0.0465 (4)	
H33A	0.716038	0.499680	0.600214	0.056*	
H33B	0.679657	0.611394	0.608577	0.056*	
C3	0.83830 (16)	0.71802 (15)	0.07895 (9)	0.0461 (3)	
C9	0.83407 (18)	0.52124 (16)	0.17324 (9)	0.0494 (4)	
H9A	0.789149	0.441714	0.194516	0.059*	
H9B	0.923813	0.557457	0.192103	0.059*	
C29	0.77116 (18)	0.59194 (16)	0.70548 (10)	0.0515 (4)	
C17	0.55484 (18)	−0.02435 (18)	0.14428 (9)	0.0518 (4)	
C20	0.34628 (19)	−0.17022 (16)	0.23234 (10)	0.0550 (4)	
H20A	0.265548	−0.187900	0.250751	0.066*	
H20B	0.376024	−0.228155	0.251938	0.066*	
C7	0.62339 (17)	0.53292 (18)	0.08302 (10)	0.0570 (4)	
H7	0.532571	0.496120	0.064282	0.068*	
C10	0.69578 (17)	0.65902 (18)	0.05044 (10)	0.0551 (4)	
H10A	0.661208	0.716286	0.064169	0.066*	
H10B	0.685028	0.645408	−0.002913	0.066*	
C27	0.94396 (19)	0.81789 (16)	0.70574 (10)	0.0550 (4)	
C28	0.82115 (19)	0.72963 (17)	0.73701 (10)	0.0547 (4)	
H28A	0.756156	0.757187	0.725515	0.066*	
H28B	0.837695	0.734841	0.790224	0.066*	
C32	0.97039 (18)	0.63053 (19)	0.60735 (10)	0.0533 (4)	
H32A	1.047882	0.685745	0.587058	0.064*	
H32B	0.941860	0.544971	0.585529	0.064*	
C26	0.91308 (17)	0.80841 (14)	0.62144 (9)	0.0480 (4)	
H26A	0.847120	0.834588	0.609658	0.058*	
H26B	0.988817	0.864055	0.599813	0.058*	
C15	0.37718 (19)	0.04088 (19)	0.14298 (10)	0.0541 (4)	
C4	0.89072 (19)	0.62571 (18)	0.05718 (10)	0.0547 (4)	
H4A	0.980834	0.662137	0.075195	0.066*	
H4B	0.882463	0.612470	0.003875	0.066*	
C16	0.50501 (19)	0.06612 (19)	0.11542 (10)	0.0560 (4)	
H16A	0.494476	0.056705	0.061962	0.067*	
H16B	0.567400	0.152538	0.131348	0.067*	
C5	0.8188 (2)	0.49778 (17)	0.08899 (10)	0.0581 (4)	
C31	0.99688 (19)	0.6358 (2)	0.69101 (11)	0.0613 (5)	
H31	1.062627	0.608260	0.703034	0.074*	
C34	1.04425 (19)	0.7718 (2)	0.72352 (11)	0.0667 (5)	
H34A	1.122481	0.826226	0.703591	0.080*	
H34B	1.063191	0.776712	0.776554	0.080*	
C18	0.4547 (2)	−0.16083 (19)	0.11936 (11)	0.0642 (5)	
H18A	0.442643	−0.173304	0.065954	0.077*	
H18B	0.484759	−0.219250	0.137810	0.077*	
C30	0.8754 (2)	0.55030 (19)	0.72313 (11)	0.0622 (5)	
H30A	0.893369	0.553496	0.776130	0.075*	
H30B	0.845513	0.463685	0.702762	0.075*	
C22	0.2800 (2)	−0.0963 (2)	0.11844 (11)	0.0669 (5)	
H22A	0.265956	−0.108476	0.065038	0.080*	
H22B	0.199032	−0.114068	0.136608	0.080*	
C11	0.9113 (2)	0.84445 (19)	0.04648 (12)	0.0637 (5)	
H11A	0.906209	0.830026	−0.006045	0.096*	
H11B	0.999280	0.882959	0.067192	0.096*	
H11C	0.874229	0.899532	0.057953	0.096*	
C6	0.6764 (2)	0.44274 (18)	0.06038 (11)	0.0675 (6)	
H6A	0.665622	0.428149	0.007073	0.081*	
H6B	0.629446	0.362181	0.080179	0.081*	
C19	0.3283 (2)	−0.18780 (18)	0.14795 (11)	0.0624 (5)	
H19	0.265373	−0.274972	0.131457	0.075*	
C37	0.7589 (2)	0.9253 (2)	0.43596 (13)	0.0725 (6)	
H37A	0.839530	0.923855	0.434650	0.109*	
H37B	0.743534	0.933165	0.486261	0.109*	
H37C	0.760564	0.996040	0.411638	0.109*	
C24	0.6820 (2)	0.0025 (3)	0.11616 (13)	0.0750 (6)	
H24A	0.716007	−0.048908	0.139055	0.112*	
H24B	0.741106	0.090204	0.128023	0.112*	
H24C	0.669158	−0.016810	0.063679	0.112*	
C39	0.1879 (3)	0.7482 (2)	0.43919 (13)	0.0772 (6)	
H39A	0.121442	0.666667	0.446964	0.116*	
H39B	0.230009	0.794210	0.485935	0.116*	
H39C	0.248720	0.737812	0.411344	0.116*	
C23	0.3304 (3)	0.1337 (3)	0.11357 (13)	0.0826 (7)	
H23A	0.390720	0.218550	0.131858	0.124*	
H23B	0.248716	0.114611	0.129763	0.124*	
H23C	0.322531	0.126083	0.060676	0.124*	
C36	0.6483 (2)	0.5065 (2)	0.73711 (14)	0.0810 (7)	
H36A	0.614309	0.422261	0.713577	0.122*	
H36B	0.586811	0.538270	0.728493	0.122*	
H36C	0.666388	0.505295	0.789161	0.122*	
C38	0.5456 (2)	0.3353 (3)	0.43141 (15)	0.0786 (6)	
H38A	0.541871	0.414355	0.430089	0.118*	
H38B	0.556891	0.318705	0.481714	0.118*	
H38C	0.467537	0.268600	0.407036	0.118*	
C35	0.9899 (3)	0.9543 (2)	0.73805 (14)	0.0888 (8)	
H35A	0.924801	0.980266	0.725943	0.133*	
H35B	1.066459	1.008865	0.717829	0.133*	
H35C	1.007226	0.959284	0.790653	0.133*	
C12	0.8726 (4)	0.4071 (3)	0.06721 (16)	0.0966 (9)	
H12A	0.860777	0.390739	0.014533	0.145*	
H12B	0.828734	0.329128	0.089069	0.145*	
H12C	0.962179	0.444489	0.084328	0.145*	

### Atomic displacement parameters (Å^2^)

**Table:**

	*U*^11^	*U*^22^	*U*^33^	*U*^12^	*U*^13^	*U*^23^
S1	0.0487 (2)	0.0458 (2)	0.0460 (2)	0.02573 (18)	0.00387 (16)	0.00512 (15)
S2	0.0469 (2)	0.0452 (2)	0.0465 (2)	0.02134 (17)	0.00555 (16)	0.00208 (15)
S3	0.0452 (2)	0.0508 (2)	0.0449 (2)	0.01982 (18)	0.00683 (16)	0.00448 (16)
O2	0.0593 (8)	0.0671 (8)	0.0715 (8)	0.0403 (7)	0.0124 (6)	0.0197 (6)
O6	0.0610 (8)	0.0462 (6)	0.0745 (9)	0.0241 (6)	−0.0017 (6)	−0.0025 (6)
O3	0.0582 (8)	0.0571 (7)	0.0800 (9)	0.0301 (6)	−0.0012 (7)	0.0199 (6)
O9	0.0518 (7)	0.0555 (7)	0.0734 (8)	0.0235 (6)	0.0180 (6)	0.0126 (6)
N2	0.0448 (7)	0.0447 (7)	0.0426 (7)	0.0218 (6)	0.0052 (6)	0.0063 (5)
N3	0.0466 (8)	0.0460 (7)	0.0408 (7)	0.0222 (6)	0.0014 (6)	0.0012 (5)
O5	0.0549 (8)	0.0566 (7)	0.0888 (10)	0.0262 (6)	−0.0052 (7)	−0.0202 (7)
O8	0.0662 (9)	0.0684 (8)	0.0819 (10)	0.0310 (7)	0.0342 (7)	0.0145 (7)
O1	0.0861 (10)	0.0835 (9)	0.0517 (7)	0.0504 (8)	0.0150 (7)	0.0031 (6)
N1	0.0524 (8)	0.0561 (8)	0.0377 (7)	0.0238 (7)	0.0040 (6)	0.0008 (6)
O4	0.1053 (12)	0.0754 (10)	0.0541 (8)	0.0353 (9)	0.0109 (8)	0.0192 (7)
O7	0.0631 (9)	0.1011 (12)	0.0585 (8)	0.0245 (8)	−0.0072 (7)	−0.0159 (8)
C1	0.0414 (8)	0.0436 (7)	0.0347 (7)	0.0188 (6)	0.0020 (6)	0.0012 (5)
C13	0.0452 (8)	0.0406 (7)	0.0389 (7)	0.0213 (6)	0.0030 (6)	0.0042 (6)
C21	0.0471 (9)	0.0523 (9)	0.0459 (8)	0.0277 (7)	0.0041 (7)	0.0049 (6)
C2	0.0418 (8)	0.0392 (7)	0.0460 (8)	0.0157 (6)	0.0002 (6)	0.0008 (6)
C25	0.0394 (8)	0.0398 (7)	0.0388 (7)	0.0199 (6)	0.0019 (6)	0.0019 (5)
C8	0.0396 (8)	0.0561 (9)	0.0469 (9)	0.0163 (7)	0.0059 (7)	0.0045 (7)
C14	0.0502 (9)	0.0506 (8)	0.0477 (8)	0.0306 (7)	0.0058 (7)	0.0076 (7)
C33	0.0417 (8)	0.0416 (8)	0.0499 (9)	0.0141 (7)	0.0029 (7)	0.0008 (6)
C3	0.0453 (8)	0.0458 (8)	0.0436 (8)	0.0175 (7)	0.0030 (6)	0.0079 (6)
C9	0.0583 (10)	0.0490 (8)	0.0482 (9)	0.0293 (8)	0.0122 (7)	0.0101 (7)
C29	0.0529 (10)	0.0469 (8)	0.0500 (9)	0.0174 (7)	0.0119 (7)	0.0068 (7)
C17	0.0551 (10)	0.0605 (10)	0.0454 (8)	0.0310 (8)	0.0075 (7)	0.0030 (7)
C20	0.0577 (10)	0.0423 (8)	0.0570 (10)	0.0159 (8)	0.0051 (8)	0.0049 (7)
C7	0.0425 (9)	0.0619 (10)	0.0463 (9)	0.0079 (8)	−0.0052 (7)	0.0014 (7)
C10	0.0501 (10)	0.0640 (10)	0.0459 (9)	0.0222 (8)	−0.0034 (7)	0.0089 (7)
C27	0.0631 (11)	0.0425 (8)	0.0474 (9)	0.0149 (8)	0.0012 (8)	−0.0042 (7)
C28	0.0646 (11)	0.0566 (10)	0.0460 (9)	0.0300 (9)	0.0108 (8)	−0.0003 (7)
C32	0.0534 (10)	0.0683 (11)	0.0518 (9)	0.0396 (9)	0.0055 (7)	0.0049 (8)
C26	0.0532 (9)	0.0369 (7)	0.0486 (9)	0.0162 (7)	0.0029 (7)	0.0024 (6)
C15	0.0604 (10)	0.0673 (11)	0.0453 (9)	0.0385 (9)	0.0020 (7)	0.0099 (7)
C4	0.0619 (11)	0.0598 (10)	0.0468 (9)	0.0292 (9)	0.0168 (8)	0.0087 (7)
C16	0.0650 (11)	0.0644 (10)	0.0447 (8)	0.0335 (9)	0.0100 (8)	0.0131 (7)
C5	0.0807 (13)	0.0510 (9)	0.0505 (9)	0.0346 (9)	0.0211 (9)	0.0042 (7)
C31	0.0606 (11)	0.0860 (13)	0.0547 (10)	0.0495 (11)	−0.0007 (8)	0.0110 (9)
C34	0.0477 (10)	0.0842 (14)	0.0510 (10)	0.0175 (10)	−0.0082 (8)	0.0000 (9)
C18	0.0839 (14)	0.0612 (11)	0.0528 (10)	0.0394 (11)	0.0032 (9)	−0.0076 (8)
C30	0.0843 (14)	0.0586 (10)	0.0519 (10)	0.0391 (10)	0.0062 (9)	0.0132 (8)
C22	0.0564 (11)	0.0861 (14)	0.0511 (10)	0.0291 (10)	−0.0084 (8)	−0.0019 (9)
C11	0.0627 (11)	0.0570 (10)	0.0664 (11)	0.0210 (9)	0.0078 (9)	0.0226 (9)
C6	0.0829 (14)	0.0473 (9)	0.0471 (9)	0.0088 (9)	0.0043 (9)	−0.0066 (7)
C19	0.0633 (11)	0.0510 (10)	0.0567 (10)	0.0146 (9)	−0.0034 (9)	−0.0094 (8)
C37	0.0709 (13)	0.0549 (11)	0.0742 (13)	0.0149 (10)	0.0018 (11)	−0.0047 (9)
C24	0.0710 (14)	0.1042 (17)	0.0612 (12)	0.0486 (13)	0.0164 (10)	0.0057 (11)
C39	0.1087 (19)	0.0742 (13)	0.0702 (13)	0.0590 (14)	0.0121 (12)	0.0177 (11)
C23	0.1070 (19)	0.1152 (19)	0.0631 (13)	0.0822 (17)	0.0089 (12)	0.0256 (12)
C36	0.0736 (15)	0.0771 (14)	0.0745 (14)	0.0141 (12)	0.0283 (12)	0.0176 (11)
C38	0.0623 (13)	0.0978 (17)	0.0868 (16)	0.0438 (13)	0.0230 (11)	0.0076 (13)
C35	0.122 (2)	0.0487 (11)	0.0681 (13)	0.0174 (12)	0.0026 (13)	−0.0133 (9)
C12	0.157 (3)	0.0811 (16)	0.0877 (17)	0.0780 (18)	0.0545 (18)	0.0141 (13)

### Geometric parameters (Å, º)

**Table:**

S1—O3	1.4497 (13)	C10—H10A	0.9700
S1—O2	1.4503 (13)	C10—H10B	0.9700
S1—O1	1.4548 (14)	C27—C34	1.527 (3)
S1—C37	1.754 (2)	C27—C35	1.531 (3)
S2—O4	1.4427 (15)	C27—C28	1.532 (3)
S2—O6	1.4478 (13)	C27—C26	1.541 (2)
S2—O5	1.4491 (14)	C28—H28A	0.9700
S2—C38	1.748 (2)	C28—H28B	0.9700
S3—O7	1.4453 (15)	C32—C31	1.528 (2)
S3—O9	1.4480 (14)	C32—H32A	0.9700
S3—O8	1.4511 (14)	C32—H32B	0.9700
S3—C39	1.751 (2)	C26—H26A	0.9700
N2—C13	1.4983 (19)	C26—H26B	0.9700
N2—H2A	0.878 (15)	C15—C16	1.531 (3)
N2—H2B	0.873 (15)	C15—C22	1.532 (3)
N2—H2C	0.896 (15)	C15—C23	1.534 (3)
N3—C25	1.5026 (19)	C4—C5	1.539 (3)
N3—H3A	0.904 (15)	C4—H4A	0.9700
N3—H3B	0.896 (15)	C4—H4B	0.9700
N3—H3C	0.890 (15)	C16—H16A	0.9700
N1—C1	1.5008 (19)	C16—H16B	0.9700
N1—H1A	0.901 (15)	C5—C12	1.528 (3)
N1—H1B	0.897 (16)	C5—C6	1.535 (3)
N1—H1C	0.893 (15)	C31—C30	1.520 (3)
C1—C9	1.524 (2)	C31—C34	1.526 (3)
C1—C2	1.525 (2)	C31—H31	0.9800
C1—C8	1.526 (2)	C34—H34A	0.9700
C13—C21	1.516 (2)	C34—H34B	0.9700
C13—C20	1.528 (2)	C18—C19	1.519 (3)
C13—C14	1.529 (2)	C18—H18A	0.9700
C21—C17	1.540 (2)	C18—H18B	0.9700
C21—H21A	0.9700	C30—H30A	0.9700
C21—H21B	0.9700	C30—H30B	0.9700
C2—C3	1.532 (2)	C22—C19	1.533 (3)
C2—H2D	0.9700	C22—H22A	0.9700
C2—H2E	0.9700	C22—H22B	0.9700
C25—C33	1.520 (2)	C11—H11A	0.9600
C25—C26	1.520 (2)	C11—H11B	0.9600
C25—C32	1.521 (2)	C11—H11C	0.9600
C8—C7	1.529 (2)	C6—H6A	0.9700
C8—H8A	0.9700	C6—H6B	0.9700
C8—H8B	0.9700	C19—H19	0.9800
C14—C15	1.538 (2)	C37—H37A	0.9600
C14—H14A	0.9700	C37—H37B	0.9600
C14—H14B	0.9700	C37—H37C	0.9600
C33—C29	1.535 (2)	C24—H24A	0.9600
C33—H33A	0.9700	C24—H24B	0.9600
C33—H33B	0.9700	C24—H24C	0.9600
C3—C4	1.533 (3)	C39—H39A	0.9600
C3—C11	1.533 (2)	C39—H39B	0.9600
C3—C10	1.533 (2)	C39—H39C	0.9600
C9—C5	1.534 (2)	C23—H23A	0.9600
C9—H9A	0.9700	C23—H23B	0.9600
C9—H9B	0.9700	C23—H23C	0.9600
C29—C36	1.527 (3)	C36—H36A	0.9600
C29—C30	1.532 (3)	C36—H36B	0.9600
C29—C28	1.533 (2)	C36—H36C	0.9600
C17—C24	1.524 (3)	C38—H38A	0.9600
C17—C16	1.530 (2)	C38—H38B	0.9600
C17—C18	1.536 (3)	C38—H38C	0.9600
C20—C19	1.532 (3)	C35—H35A	0.9600
C20—H20A	0.9700	C35—H35B	0.9600
C20—H20B	0.9700	C35—H35C	0.9600
C7—C6	1.520 (3)	C12—H12A	0.9600
C7—C10	1.530 (3)	C12—H12B	0.9600
C7—H7	0.9800	C12—H12C	0.9600
			
O3—S1—O2	112.16 (8)	C27—C28—H28B	109.3
O3—S1—O1	112.49 (9)	C29—C28—H28B	109.3
O2—S1—O1	112.24 (9)	H28A—C28—H28B	107.9
O3—S1—C37	106.48 (10)	C25—C32—C31	108.64 (14)
O2—S1—C37	106.90 (11)	C25—C32—H32A	110.0
O1—S1—C37	106.03 (11)	C31—C32—H32A	110.0
O4—S2—O6	112.33 (9)	C25—C32—H32B	110.0
O4—S2—O5	112.53 (10)	C31—C32—H32B	110.0
O6—S2—O5	111.99 (8)	H32A—C32—H32B	108.3
O4—S2—C38	106.23 (12)	C25—C26—C27	109.43 (13)
O6—S2—C38	107.04 (11)	C25—C26—H26A	109.8
O5—S2—C38	106.18 (11)	C27—C26—H26A	109.8
O7—S3—O9	112.24 (10)	C25—C26—H26B	109.8
O7—S3—O8	112.80 (10)	C27—C26—H26B	109.8
O9—S3—O8	111.98 (8)	H26A—C26—H26B	108.2
O7—S3—C39	106.65 (12)	C16—C15—C22	108.80 (16)
O9—S3—C39	105.98 (11)	C16—C15—C23	110.50 (17)
O8—S3—C39	106.65 (11)	C22—C15—C23	111.15 (18)
C13—N2—H2A	108.9 (13)	C16—C15—C14	108.38 (14)
C13—N2—H2B	108.9 (13)	C22—C15—C14	108.47 (15)
H2A—N2—H2B	108.3 (18)	C23—C15—C14	109.49 (16)
C13—N2—H2C	107.2 (13)	C3—C4—C5	111.38 (15)
H2A—N2—H2C	109.0 (18)	C3—C4—H4A	109.4
H2B—N2—H2C	114.4 (18)	C5—C4—H4A	109.4
C25—N3—H3A	111.5 (13)	C3—C4—H4B	109.4
C25—N3—H3B	111.5 (13)	C5—C4—H4B	109.4
H3A—N3—H3B	105.7 (18)	H4A—C4—H4B	108.0
C25—N3—H3C	111.2 (13)	C17—C16—C15	111.81 (15)
H3A—N3—H3C	105.8 (18)	C17—C16—H16A	109.3
H3B—N3—H3C	110.8 (19)	C15—C16—H16A	109.3
C1—N1—H1A	106.7 (14)	C17—C16—H16B	109.3
C1—N1—H1B	107.8 (14)	C15—C16—H16B	109.3
H1A—N1—H1B	113 (2)	H16A—C16—H16B	107.9
C1—N1—H1C	107.3 (14)	C12—C5—C9	109.66 (18)
H1A—N1—H1C	112.9 (19)	C12—C5—C6	110.9 (2)
H1B—N1—H1C	109 (2)	C9—C5—C6	108.75 (16)
N1—C1—C9	108.80 (13)	C12—C5—C4	110.77 (18)
N1—C1—C2	109.46 (12)	C9—C5—C4	108.30 (15)
C9—C1—C2	110.08 (13)	C6—C5—C4	108.38 (16)
N1—C1—C8	108.44 (13)	C30—C31—C34	109.47 (17)
C9—C1—C8	110.26 (14)	C30—C31—C32	110.15 (16)
C2—C1—C8	109.76 (13)	C34—C31—C32	108.73 (16)
N2—C13—C21	109.02 (12)	C30—C31—H31	109.5
N2—C13—C20	108.75 (13)	C34—C31—H31	109.5
C21—C13—C20	110.00 (14)	C32—C31—H31	109.5
N2—C13—C14	108.56 (12)	C31—C34—C27	110.87 (15)
C21—C13—C14	110.25 (13)	C31—C34—H34A	109.5
C20—C13—C14	110.23 (14)	C27—C34—H34A	109.5
C13—C21—C17	109.86 (13)	C31—C34—H34B	109.5
C13—C21—H21A	109.7	C27—C34—H34B	109.5
C17—C21—H21A	109.7	H34A—C34—H34B	108.1
C13—C21—H21B	109.7	C19—C18—C17	110.38 (15)
C17—C21—H21B	109.7	C19—C18—H18A	109.6
H21A—C21—H21B	108.2	C17—C18—H18A	109.6
C1—C2—C3	110.01 (12)	C19—C18—H18B	109.6
C1—C2—H2D	109.7	C17—C18—H18B	109.6
C3—C2—H2D	109.7	H18A—C18—H18B	108.1
C1—C2—H2E	109.7	C31—C30—C29	110.54 (15)
C3—C2—H2E	109.7	C31—C30—H30A	109.5
H2D—C2—H2E	108.2	C29—C30—H30A	109.5
N3—C25—C33	109.30 (12)	C31—C30—H30B	109.5
N3—C25—C26	108.86 (12)	C29—C30—H30B	109.5
C33—C25—C26	109.96 (13)	H30A—C30—H30B	108.1
N3—C25—C32	108.23 (13)	C15—C22—C19	110.50 (15)
C33—C25—C32	110.02 (13)	C15—C22—H22A	109.5
C26—C25—C32	110.44 (14)	C19—C22—H22A	109.5
C1—C8—C7	108.38 (14)	C15—C22—H22B	109.5
C1—C8—H8A	110.0	C19—C22—H22B	109.5
C7—C8—H8A	110.0	H22A—C22—H22B	108.1
C1—C8—H8B	110.0	C3—C11—H11A	109.5
C7—C8—H8B	110.0	C3—C11—H11B	109.5
H8A—C8—H8B	108.4	H11A—C11—H11B	109.5
C13—C14—C15	109.31 (13)	C3—C11—H11C	109.5
C13—C14—H14A	109.8	H11A—C11—H11C	109.5
C15—C14—H14A	109.8	H11B—C11—H11C	109.5
C13—C14—H14B	109.8	C7—C6—C5	110.54 (14)
C15—C14—H14B	109.8	C7—C6—H6A	109.5
H14A—C14—H14B	108.3	C5—C6—H6A	109.5
C25—C33—C29	110.04 (13)	C7—C6—H6B	109.5
C25—C33—H33A	109.7	C5—C6—H6B	109.5
C29—C33—H33A	109.7	H6A—C6—H6B	108.1
C25—C33—H33B	109.7	C18—C19—C20	109.84 (16)
C29—C33—H33B	109.7	C18—C19—C22	109.75 (18)
H33A—C33—H33B	108.2	C20—C19—C22	108.95 (17)
C2—C3—C4	108.56 (13)	C18—C19—H19	109.4
C2—C3—C11	110.40 (14)	C20—C19—H19	109.4
C4—C3—C11	110.30 (15)	C22—C19—H19	109.4
C2—C3—C10	108.42 (14)	S1—C37—H37A	109.5
C4—C3—C10	108.78 (15)	S1—C37—H37B	109.5
C11—C3—C10	110.32 (14)	H37A—C37—H37B	109.5
C1—C9—C5	109.68 (14)	S1—C37—H37C	109.5
C1—C9—H9A	109.7	H37A—C37—H37C	109.5
C5—C9—H9A	109.7	H37B—C37—H37C	109.5
C1—C9—H9B	109.7	C17—C24—H24A	109.5
C5—C9—H9B	109.7	C17—C24—H24B	109.5
H9A—C9—H9B	108.2	H24A—C24—H24B	109.5
C36—C29—C30	111.16 (18)	C17—C24—H24C	109.5
C36—C29—C28	110.63 (17)	H24A—C24—H24C	109.5
C30—C29—C28	108.68 (16)	H24B—C24—H24C	109.5
C36—C29—C33	109.98 (16)	S3—C39—H39A	109.5
C30—C29—C33	107.91 (15)	S3—C39—H39B	109.5
C28—C29—C33	108.40 (14)	H39A—C39—H39B	109.5
C24—C17—C16	110.87 (17)	S3—C39—H39C	109.5
C24—C17—C18	110.44 (17)	H39A—C39—H39C	109.5
C16—C17—C18	108.76 (15)	H39B—C39—H39C	109.5
C24—C17—C21	110.12 (15)	C15—C23—H23A	109.5
C16—C17—C21	108.09 (14)	C15—C23—H23B	109.5
C18—C17—C21	108.49 (15)	H23A—C23—H23B	109.5
C13—C20—C19	108.48 (14)	C15—C23—H23C	109.5
C13—C20—H20A	110.0	H23A—C23—H23C	109.5
C19—C20—H20A	110.0	H23B—C23—H23C	109.5
C13—C20—H20B	110.0	C29—C36—H36A	109.5
C19—C20—H20B	110.0	C29—C36—H36B	109.5
H20A—C20—H20B	108.4	H36A—C36—H36B	109.5
C6—C7—C8	109.66 (16)	C29—C36—H36C	109.5
C6—C7—C10	109.48 (17)	H36A—C36—H36C	109.5
C8—C7—C10	109.83 (15)	H36B—C36—H36C	109.5
C6—C7—H7	109.3	S2—C38—H38A	109.5
C8—C7—H7	109.3	S2—C38—H38B	109.5
C10—C7—H7	109.3	H38A—C38—H38B	109.5
C7—C10—C3	110.09 (14)	S2—C38—H38C	109.5
C7—C10—H10A	109.6	H38A—C38—H38C	109.5
C3—C10—H10A	109.6	H38B—C38—H38C	109.5
C7—C10—H10B	109.6	C27—C35—H35A	109.5
C3—C10—H10B	109.6	C27—C35—H35B	109.5
H10A—C10—H10B	108.2	H35A—C35—H35B	109.5
C34—C27—C35	111.57 (19)	C27—C35—H35C	109.5
C34—C27—C28	108.39 (16)	H35A—C35—H35C	109.5
C35—C27—C28	110.15 (18)	H35B—C35—H35C	109.5
C34—C27—C26	109.22 (16)	C5—C12—H12A	109.5
C35—C27—C26	109.83 (16)	C5—C12—H12B	109.5
C28—C27—C26	107.59 (15)	H12A—C12—H12B	109.5
C27—C28—C29	111.83 (14)	C5—C12—H12C	109.5
C27—C28—H28A	109.3	H12A—C12—H12C	109.5
C29—C28—H28A	109.3	H12B—C12—H12C	109.5
			
N2—C13—C21—C17	−179.89 (13)	C35—C27—C26—C25	−179.97 (18)
C20—C13—C21—C17	60.96 (17)	C28—C27—C26—C25	−60.08 (19)
C14—C13—C21—C17	−60.81 (17)	C13—C14—C15—C16	−58.79 (19)
N1—C1—C2—C3	179.87 (13)	C13—C14—C15—C22	59.17 (19)
C9—C1—C2—C3	60.33 (17)	C13—C14—C15—C23	−179.39 (18)
C8—C1—C2—C3	−61.22 (17)	C2—C3—C4—C5	59.02 (19)
N1—C1—C8—C7	−179.81 (14)	C11—C3—C4—C5	−179.90 (16)
C9—C1—C8—C7	−60.78 (18)	C10—C3—C4—C5	−58.78 (19)
C2—C1—C8—C7	60.66 (18)	C24—C17—C16—C15	180.00 (16)
N2—C13—C14—C15	−179.95 (14)	C18—C17—C16—C15	58.4 (2)
C21—C13—C14—C15	60.69 (18)	C21—C17—C16—C15	−59.2 (2)
C20—C13—C14—C15	−60.94 (18)	C22—C15—C16—C17	−58.15 (19)
N3—C25—C33—C29	179.97 (13)	C23—C15—C16—C17	179.57 (17)
C26—C25—C33—C29	−60.58 (17)	C14—C15—C16—C17	59.6 (2)
C32—C25—C33—C29	61.26 (17)	C1—C9—C5—C12	−179.83 (19)
C1—C2—C3—C4	−58.56 (18)	C1—C9—C5—C6	−58.40 (19)
C1—C2—C3—C11	−179.59 (14)	C1—C9—C5—C4	59.2 (2)
C1—C2—C3—C10	59.46 (17)	C3—C4—C5—C12	−179.67 (19)
N1—C1—C9—C5	179.41 (14)	C3—C4—C5—C9	−59.4 (2)
C2—C1—C9—C5	−60.65 (18)	C3—C4—C5—C6	58.44 (19)
C8—C1—C9—C5	60.60 (18)	C25—C32—C31—C30	59.1 (2)
C25—C33—C29—C36	178.98 (17)	C25—C32—C31—C34	−60.8 (2)
C25—C33—C29—C30	−59.61 (18)	C30—C31—C34—C27	−60.0 (2)
C25—C33—C29—C28	57.92 (18)	C32—C31—C34—C27	60.4 (2)
C13—C21—C17—C24	−179.97 (16)	C35—C27—C34—C31	179.94 (18)
C13—C21—C17—C16	58.77 (18)	C28—C27—C34—C31	58.5 (2)
C13—C21—C17—C18	−59.00 (18)	C26—C27—C34—C31	−58.5 (2)
N2—C13—C20—C19	−179.97 (15)	C24—C17—C18—C19	179.54 (17)
C21—C13—C20—C19	−60.65 (19)	C16—C17—C18—C19	−58.6 (2)
C14—C13—C20—C19	61.13 (19)	C21—C17—C18—C19	58.8 (2)
C1—C8—C7—C6	60.18 (19)	C34—C31—C30—C29	59.6 (2)
C1—C8—C7—C10	−60.2 (2)	C32—C31—C30—C29	−59.9 (2)
C6—C7—C10—C3	−60.18 (19)	C36—C29—C30—C31	179.75 (18)
C8—C7—C10—C3	60.3 (2)	C28—C29—C30—C31	−58.3 (2)
C2—C3—C10—C7	−59.00 (19)	C33—C29—C30—C31	59.08 (19)
C4—C3—C10—C7	58.89 (19)	C16—C15—C22—C19	57.9 (2)
C11—C3—C10—C7	180.00 (16)	C23—C15—C22—C19	179.75 (18)
C34—C27—C28—C29	−58.18 (19)	C14—C15—C22—C19	−59.8 (2)
C35—C27—C28—C29	179.51 (18)	C8—C7—C6—C5	−60.2 (2)
C26—C27—C28—C29	59.8 (2)	C10—C7—C6—C5	60.35 (19)
C36—C29—C28—C27	−179.52 (17)	C12—C5—C6—C7	179.38 (17)
C30—C29—C28—C27	58.2 (2)	C9—C5—C6—C7	58.7 (2)
C33—C29—C28—C27	−58.9 (2)	C4—C5—C6—C7	−58.82 (19)
N3—C25—C32—C31	−179.24 (15)	C17—C18—C19—C20	−60.1 (2)
C33—C25—C32—C31	−59.88 (19)	C17—C18—C19—C22	59.7 (2)
C26—C25—C32—C31	61.68 (19)	C13—C20—C19—C18	60.0 (2)
N3—C25—C26—C27	−178.60 (14)	C13—C20—C19—C22	−60.3 (2)
C33—C25—C26—C27	61.69 (18)	C15—C22—C19—C18	−59.5 (2)
C32—C25—C26—C27	−59.91 (18)	C15—C22—C19—C20	60.8 (2)
C34—C27—C26—C25	57.37 (19)		

### Hydrogen-bond geometry (Å, º)

**Table:**

*D*—H···*A*	*D*—H	H···*A*	*D*···*A*	*D*—H···*A*
N1—H1*A*···O7^i^	0.90 (2)	1.92 (2)	2.819 (2)	177 (2)
N1—H1*B*···O1	0.90 (2)	1.94 (2)	2.833 (2)	179 (2)
N1—H1*C*···O4	0.89 (2)	1.96 (2)	2.844 (2)	170 (2)
N2—H2*A*···O2^ii^	0.88 (2)	1.92 (2)	2.7991 (18)	179 (2)
N2—H2*B*···O9^ii^	0.87 (2)	1.94 (2)	2.8090 (19)	175 (2)
N2—H2*C*···O6	0.90 (2)	1.90 (2)	2.7923 (19)	177 (2)
N3—H3*A*···O3	0.90 (2)	1.91 (2)	2.7717 (19)	159 (2)
N3—H3*B*···O5	0.90 (2)	1.89 (2)	2.7752 (19)	172 (2)
N3—H3*C*···O8^i^	0.89 (2)	1.90 (2)	2.785 (2)	172 (2)
C39—H39*B*···O6^iii^	0.96	2.59	3.423 (3)	145

Symmetry codes: (i) *x*+1, *y*, *z*; (ii) *x*, *y*−1, *z*; (iii) −*x*+1, −*y*+1, −*z*+1.
