# Supplementary figures and images for: (3,5-Di­methyl­adamantan-1-yl)ammonium methane­sulfonate (memanti­nium mesylate): synthesis, structure and solid-state properties
Source: Acta Crystallogr E Crystallogr Commun. 2019 Jul 26;75(Pt 8):1274–9. doi: 10.1107/S2056989019009988 (PMC6690476; doi:10.1107/S2056989019009988)

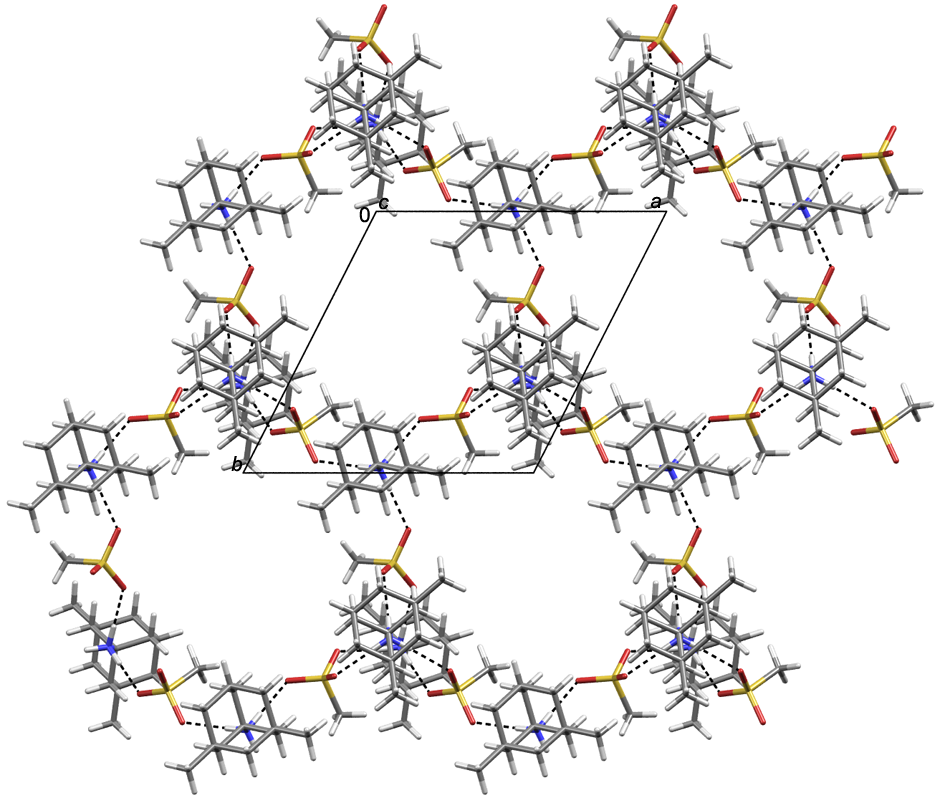

Supplement: Supplementary file 3 [file e-75-01274-sup3.tif]
